# Anatomical versus functional classification of ankyloglossia and their association with temporomandibular joint disorders in adults: a cross sectional study

**DOI:** 10.22514/jofph.2025.049

**Published:** 2025-09-12

**Authors:** Anita Beckmann, Ingrid Peroz, Simon Peroz

**Affiliations:** ^1^Department of Prosthodontics, Geriatric Dentistry and Craniomandibular Function, University Medicine Berlin, 14197 Berlin, BE, Germany

**Keywords:** Tongue-tie, Tethered oral tissue, Ankyloglossia, Restrictive tongue movement, Temporomandibular disorders (TMD)

## Abstract

**Background**: Restrictive lingual frenula, commonly referred to as ankyloglossia, are well documented in pediatric literature, with established impacts on breastfeeding, swallowing, reflux, speech, maxillary development, breathing, and sleep in children. However, data on its effects in adolescents and adults remains limited. This study aimed to investigate the correlation between restrictive lingual frenula and the development of temporomandibular disorders (TMD) in an adult population. **Methods**: A total of 129 patients (aged 18–80 years; 41 males and 88 females) were assessed for TMD signs and symptoms using the three Lövgren screening questions, supplemented by a clinical examination following the German Association for Craniomandibular Function and Diagnostics (DGFDT) screening protocol. The presence and severity of ankyloglossia were evaluated using the Tongue Range of Motion Ratio (TRMR 2019) and Kotlow’s free tongue classification. Association between variables were analyzed using Chi-square tests. **Results**: TMD was identified in 49.1% of the cohort, while ankyloglossia was observed in 46.7% based on the TRMR. No significant association was found between TMD and either anterior (39.6%) or posterior (34.9%) tongue restriction using the TRMR criteria. However, when categorized according to Kotlow’s classification, mild to severe ankyloglossia (59.2%) showed a statistically significant association with TMD (*p* = 0.026). Age and sex were not significantly associated with the presence of ankyloglosson, while TMD occurrence was found to be correlated with sex. **Conclusions**: A weak but significant correlation was observed between TMD and the degree of ankyloglossia, particularly when assessed using Kotlow’s method. Further studies with larger sample sizes, stratified by age and sex, incorporating occlusal factors, and employing a standardized validated assessment tool adapted for adults, accounting for both anatomical and functional criteria are warranted to explore potential causal relationships.

## 1. Introduction

The lingual frenulum is a stratified bundle of submucosal connective tissue that connects the midline of the underside of the tongue to the floor of the mouth, where it spans horizontally, with considerable individual variability observed in the proportion and distribution of collagen and elastin fibers within this structure. A congenitally restricted lingual frenulum known as ankyloglossia or tethered oral tissue, can be diagnosed in approximately 2.5% to 46.3% of newborns [1], and the prevalence varies largely due to variations in classification systems and differences across populations [2]. Ankyloglossia has been associated with a range of functional impairments, including difficulties with breastfeeding [1, 3, 4], swallowing [5], gastroesophageal reflux [6], mouth breathing and obstructive sleep apnea (OSA) [7], speech and articulation [5, 8, 9], eating difficulties [8], dental malocclusion [5, 6, 8, 10], social embarrassment [8], oral hygiene issues [8], chewing muscle activity [11], craniofacial development [11], and hyoid position [11]. While most of the literature focuses on infants and children, studies investigating the prevalence and consequences of ankyloglossia in adolescents and adults are limited, with many of the aforementioned symptoms being either directly or indirectly related to tongue function or position [12]. The tongue, supported by its intrinsic and extrinsic musculature and its anatomical connections to mandible and hyoid bone, plays a critical role in the masticatory system [11, 13].

Despite the clinical relevance of ankyloglossia, there are no universally accepted objective diagnostic criteria for its identification or severity grading. As noted by Cordray *et al*. [8], both validated and unvalidated manual and visual assessment methods exist, ranging in standardization and reliability. One commonly employed anatomical classification is that proposed by Coryllos [4], which is based on palpation to distinguish between anterior and the often-overlooked posterior forms of ankyloglossia. Kotlow introduced a straightforward anatomical method that categorizes the condition as complete, severe, moderate, or mild based on the measured length of the free tongue [14]. Comparatively the Hazelbaker Assessment Tool adopts a functional approach, incorporating five appearance-related and seven function-related items [15]. Although it is reported to have high validity and reliability, its correlation with breastfeeding difficulties, the most frequent indication for frenotomy, remains inconsistent [16]. Another validated classification, proposed by Zaghi *et al*. [17], is functionally oriented and assesses tongue mobility through quantifiable measurements of anterior and posterior tongue movements, which have been shown to correlate well with patients’ self-reported functional limitations.

Temporomandibular disorders (TMD) include muscle and/or joint pain and dysfunction of the temporomandibular joint (TMJ) system. Globally, the incidence of TMD is approximately 34%, with a higher prevalence among females, particularly in South America, with age identified as also playing a role with the highest incidence observed in individuals aged 18 to 60 years [18]. The etiology of TMD is multifactorial, involving genetic predisposition, trauma, bruxism, psychological disorders, and occlusal abnormalities, although the relevance of malocclusion remains controversial [19, 20, 21, 22, 23].

The potential relationship between ankyloglossia and TMD has not yet been systematically explored. However, it may be hypothesized based on known physiological and developmental interrelations. Orofacial growth is influenced by tongue posture and activity, particularly its position high in the palate and its role in sucking, swallowing, and mastication. A shortened lingual frenulum may reduce these stimuli, potentially leading to orthodontic alterations such as crossbite or disproportionate growth of the maxilla and mandible [11]. These structural changes may result in a retruded lower jaw position, which could predispose individuals to TMD.

In addition, a short lingual frenulum has been associated with an increased risk of sleep-related breathing disorders, including sleep apnea [7, 8]. Patients with obstructive sleep apnea (OSA) often exhibit a posteriorly positioned mandibular condyle, which can be confirmed through Cone Beam Computed Tomography (CBCT) and Magnetic Resonance Imaging (MRI) [24]. OSA is strongly linked with sleep bruxism, which in turn is a known risk factor for TMD development [20], suggesting a possible indirect association between ankyloglossia and TMD.

Moreover, the lingual frenulum interacts with muscles connected to the hyoid bone, as well as with the masticatory and facial muscles [25]. This anatomical and functional relationship may contribute to elevated muscle activity and myogenous TMD [11]. Additional evidence supports this connection: patients with TMD have been found to exhibit restricted tongue mobility in functional tasks such as tongue pressure, protrusion, and spontaneous swallowing [26]. Furthermore, occlusal therapy for TMD has been shown to influence hyoid position. In a controlled clinical trial, Derwich and Pawlowska reported that treatment with relaxation splint and physiotherapy led to a lowered hyoid position and a reduction in oropharyngeal space, a finding associated with increased risk of OSA [27].

Based on the above considerations, this study hypothesizes that restricted tongue mobility is associated with a higher prevalence of TMD.

## 2. Materials and methods

This study was approved by the ethics committee of the Charité. All participants provided written informed consent before enrollment. Patient recruitment was conducted at a private dental practice specializing in TMD and ankyloglossia between March 2022 and March 2023. Participants were recruited into this study if they were over 18 years of age and excluded if they had a history of TMJ surgery, mental illness, or language deficits that would impair the ability to follow instructions or questions. A total of 129 patients participated in the study, comprising 41 males (31.8%) and 88 females (68.2%). The age of participants ranged from 18 to 79 years, with a mean age of 44.1 ± 11.4 years.

### 2.1 TMD Screening and clinical examination

To screen for TMD in adults, the three question tool developed by Lövgren *et al*. [28] was used due to its validity and cost-effectiveness. The questions were as follows:

Q1: Do you experience pain in your temple, face, jaw or jaw joint once a week or more?

Q2: Do you have pain once a week or more when opening your mouth or chewing?

Q3: Does your jaw lock or become stuck once a week or more?

In addition to the questionnaire, clinical examinations were performed according to standardized diagnostic criteria:

E1: Palpation of the temporalis and masseter muscles, following the Diagnostic Criteria for Temporomandibular Disorders (DC/TMD) guidelines [29].

E2: Preauricular and intraauricular palpation of the TMJ, also according to DC/TMD guidelines [29].

E3: Measurement of the maximal interincisal mouth opening (MIO).

Participants who answered “yes” to at least one of the three screening questions, exhibited pain on palpation of at least one muscle or one TMJ on either side, or had a maximal interincisal opening less than 38 mm were classified as probable TMD patients.

### 2.2 Assessment and grading of ankyloglossia

Tongue-mobility was assessed using the Tongue Range of Motion Ratio (TRMR) protocol published in 2019 by Zaghi *et al*. [17]. MIO was measured with a ruler placed between the upper and lower right central incisors. This measurement was then repeated while the tip of the tongue was placed at the maxillary incisive papilla (MIO-TIP), presenting anterior tongue mobility.

To evaluate posterior tongue mobility, the MIO was measured while the patient performed a tongue-suction maneuver, in which the posterior tongue was elevated to the roof palate in a movement similar to tongue clicking (MIO-sucked). In this position at least the anterior third of the tongue was required to make contact with the palate. If a patient was unable to perform the suction maneuver, the MIO-sucked value was recorded as 0. The TRMR values were then calculated as percentages:

TRMR-TIP (%) = MIO-TIP × 100/MIO

TRMR-sucked (%) = MIO-sucked × 100/MIO

The TRMR values were used to classify the severity of ankyloglossia, as shown in Table 1 (Ref. [17]). Grades 2d, 3 and 4 were considered indicative of restricted tongue mobility, while grade 1, 2a, 2b and 2c were classified as non-restricted tongue mobility.

**Table 1.** Classification of ankyloglossia by Zaghi *et al*. [17].

Grade	Description	Anterior tongue mobility	Posterior tongue mobility
1	Significantly above average	>80%	>60%
2a	Above average	70–80%	50–60%
2b	Slightly above average	65–70%	45–50%
2c	Average	60 ± 5%	40 ± 5%
2d	Slightly below average	50–55%	30–35%
3	Below average	25–50%	5–30%
4	Significantly below average	<25%	<5% or impossible

### 2.3 Anatomical classification of ankyloglossia

Ankyloglossia was evaluated using the classification proposed by Kotlow *et al*. [14]. The free-tongue length was measured with a Boley gauge, determining the distance between the tip of the tongue and the point of insertion of the lingual frenulum at the base of the tongue. During the measurement, a dental instrument was used to gently place the insertion point in a position approximating the tip of the tongue, thereby ensuring consistent and accurate readings. The grading criteria are summarized in Table 2 (Ref. [14]). A free tongue length of less than 16 mm was interpreted as indicative of restricted tongue mobility.

**Table 2.** Classification of ankyloglossia by Kotlow *et al*. [14].

Classification of Ankyloglossia	Description	Free tongue distance
0	Normal range	>16 mm
1	Mild ankyloglossia	12–16 mm
2	Moderate ankyloglossia	8–11 mm
3	Severe ankyloglossia	3–7 mm
4	Complete ankyloglossia	<3 mm

### 2.4 Statistical analysis

All statistical analyses were performed using IBM SPSS Statistics software, version 30.1 (IBM Corp., Armonk, NY, USA). Descriptive statistics were presented in the form of cross tables. The TRMR measurements, reflecting anterior and posterior tongue mobility according to Zaghi *et al*. [17] protocols, were used to assess restriction levels. To evaluate the association between TMD and ankyloglossia classifications (both Zaghi or Kotlow), as well as patient sex and age, comparative analyses were conducted using the Chi-square (χ^2^) test, as the variables under investigation were all categorical. A statistically significant correlation between two variables was defined as a distribution of observed frequencies that significantly deviated from the expected frequencies under the null hypothesis. A *p*-value of less than 0.05 was considered statistically significant. To assess the influence of age, participants were stratified into two groups: those younger than 40 years and those aged 40 years or older.

## 3. Results

### 3.1 Incidence of TMD and ankyloglossia

Based on the Lövgren screening questionnaire [28], in combination with clinical examination 114 of the 129 patients were classified as probable TMD cases. The occurrence of TMD was significantly higher in females compared to males (χ^2^-test, *p* = 0.013; Table 3).

**Table 3.** Incidence of probable TMD in males and females.

	Males	Females
No TMD	9 (22.0%)	6 (6.8%)
Probable TMD	32 (78.0%)	82 (93.2%)
Total	41 (100.0%)	88 (100.0%)

*χ^2^-test, p = 0.013. TMD: temporomandibular disorders.*

Using the TRMR classification by Zaghi *et al*. [17], 51 patients (39.6%) had anterior tongue restriction, indicated by a below-average TRMR-TIP value, and 45 patients (34.9%) had posterior tongue restriction, indicated by a below-average TRMR-sucked value (Table 4, Ref. [17]). In total, 63 patients (48.8%) exhibited either anterior and/or posterior tongue restriction. No significant correlation was found between the presence of ankyloglossia classified by TRMR and age (χ^2^-test, *p* = 0.278) or sex (χ^2^-test, *p* = 0.060).

**Table 4.** Incidence of TRMR classification by Zaghi *et al*. [17].

Grade/Description	TRMR-TIP N (%)	TRMR-sucked N (%)	Sex
1/Significant above average	29 (22.5%)	14 (10.8%)	16 males (39.0%) 50 females (56.8%)
2a/Above average	17 (13.2%)	20 (15.5%)	
2b/Slightly above average	13 (10.1%)	21 (16.3%)	
2c/Average	19 (14.7%)	29 (22.5%)	
2d/Slightly below average	9 (7.0%)	7 (5.4%)	25 males (61.0%) 38 females (43.2%)
3/Below average	36 (27.9%)	21 (16.3%)	
4/Significant below average	6 (4.7%)	17 (13.2%)	
Total	129 (100%)	129 (100%)	

*TRMR-TIP (%) = MIO-TIP × 100/MIO; TRMR-sucked (%) = MIO-sucked × 100/MIO. TRMR-TIP: Tongue Range of Motion Ratio with tip of the tongue placed at the maxillary incisive papilla; TRMR-sucked: Tongue Range of Motion Ratio with tongue sucked.*

The free tongue measurement according to Kotlow *et al*. [14] was performed in 128 patients, with values ranging from 4 mm to 28 mm. According to this classification, 76% of patients were diagnosed with mild, moderate, or severe ankyloglossia; none had complete ankyloglossia (Table 5, Ref. [14]). In addition, we observed no significant correlation between Kotlow-defined ankyloglossia and age (χ^2^-test, *p* = 0.116) or sex (χ^2^-test, *p* = 0.523).

**Table 5.** Incidence of ankyloglossia as defined by Kotlow *et al*. [14].

Free tongue measurement (mm)	N = 128	%	Sex
0. Normal >16 mm	52	40.2	15 males (36.6%) 37 females (42.5%)
1. Mild: 12–16 mm	44	34.6	26 males (63.4%) 50 females (57.5%)
2. Moderate: 8–11 mm	27	21.3	
3. Severe: 3–7 mm	5	3.9	
4. Complete: 0–3 mm	0	0	

### 3.2 Association between TMD and ankyloglossia severity

On evaluating the incidence of TMD in relation to TRMR-TIP and TRMR-sucked measurements based on the classification by Zaghi *et al*. [17], the χ^2^ test revealed no significant correlation, neither between TMD and anterior restriction, nor between TMD and posterior restriction, nor between TMD and the presence of anterior and/or posterior restricted frenulum (χ^2^-test, *p* = 0.918; Table 6 (Ref. [17])).

**Table 6.** Correlation between Zaghi *et al*.’s [17] classification of restricted frenulum (anterior and/or posterior) and TMD.

Anterior and/or posterior restrictions, N (%)				
		Yes	No	Sum
TMD n (%)	Yes	8 (53.3%)	7 (46.7%)	15
	No	58 (50.9%)	56 (49.1%)	114
	Sum	66	63	129

*χ^2^-test, p = 0.918. TMD: temporomandibular disorders.*

In contrast, when using Kotlow’s classification, the presence of mild, moderate, or severe ankyloglossia was significantly associated with TMD (χ^2^-test, *p* = 0.026; Table 7 (Ref. [14])).

**Table 7.** Correlation between Kotlow-defined ankyloglossia classification and TMD.

		TMD N (%)	
		Yes	No
Classification of Ankyloglossia according to Kotlow [14]	0 (Normal)	41 (36.6%)	10 (66.6%)
	1 (Mild)	43 (38.4%)	1 (6.7%)
	2 (Moderate)	24 (21.4%)	3 (20.0%)
	3 (Severe)	4 (3.6%)	1 (6.7%)
	4 (Complete)	0 (0.0%)	0 (0.0%)

*χ^2^-test, p = 0.026. TMD: Temporomandibular Disorders.*

## 4. Discussion

### 4.1 Study strengths and limitations

The consecutively recruited participants represent a clinical population from a practice with a specific focus on TMD and ankyloglossia, which may likely explain the high incidence of both conditions observed in the present cohort but limits the generalizability of the findings to broader populations.

Several limitations must be considered. First the sample size was relatively small. A formal power analysis was not performed as the study was designed as a pilot investigation.

Second, TMD screening was conducted using the three Lövgren questions, which are validated and appropriate for use in primary dental healthcare. The tool has a specificity of 82% and a sensitivity of 79%. For at least one affirmative answer, it shows a low positive predictive value of 30% but a high negative predictive value of 97% [28]. These predictive values are highly dependent on TMD prevalence. In a specialized setting, the positive predictive value increases to 79%, while the negative predictive value decreases to 81% [28]. Given the specialized context of the present study, it can be assumed that probable TMD patients were identified with high probability.

Third, in addition to the anamnestic data, clinical examinations were performed to identify symptom-free functional disorders of the temporalis and/or masseter muscles, myogenous or arthrogenous limitations of the mandibular mobility, as well as capsulitis and/or synovitis. However, malocclusion was not included in the clinical assessment, despite the potential for ankyloglossia to influence jaw development and therefore occlusion. This is a limitation that should be recognized and addressed in future studies.

Fourth, all patients included in this study were older than 18 years. Information regarding potential corrections of developmental disorders such as the previous orthodontic, orthopedic, or prosthetic treatments was not collected. These factors may have influenced both tongue mobility and TMD symptoms.

Fifth, although the association between TMD and occlusion remains under ongoing discussion, with several studies reporting a weak or no connection [22], the exclusion of occlusal assessment in this study limits the ability to explore this relationship further.

Lastly, since there is currently no internationally accepted, validated diagnostic tool for ankyloglossia, we depended on the anatomical classification by Kotlow *et al*. [14] and the functional TRMR classification by Zaghi *et al*. [17] to evaluate the presence and severity of tongue restriction. However, it should be noted that all the measurements were performed by the same well-trained examiner to ensure consistency and enhance reliability.

### 4.2 Interpretation of associations between ankyloglossia and TMD

To the best of our knowledge, no previous studies have specifically examined the correlation between restricted tongue mobility and TMD, and existing studies have primarily focused on frenoplasty techniques and their effects on sleep-related breathing disorders or breastfeeding outcomes [3, 6, 7, 8, 16, 25, 30, 31]. In the present study, while the visible classification of ankyloglossia according to Kotlow showed a significant correlation with TMD, the functional classification based on the TRMR protocol by Zaghi did not support this association. We believe that these differing results could be explained by the fundamental differences between the classification approaches.

Consistent with findings from other studies, a higher prevalence of probable TMD was observed in female patients [18, 28]. The association between TMD and age narrowly missed statistical significance (*p* = 0.060), likely due to the limited sample size, although patients were stratified into only two cohorts, those younger than 40 years and those aged 40 years or older.

#### 4.2.1 Limitations of the functional TRMR classification

Zaghi’s classification can be used to distinguish between two types of tongue restriction: anterior and posterior. In the present study, we applied a dichotomous approach by combining anterior and/or posterior restrictions in the analysis. Contrary to our expectation, this did not result in a higher probability of detecting a significant association with TMD. Furthermore, no correlation was found between restricted tongue mobility and either age or sex.

The functional TRMR measurement protocol developed by Zaghi in 2019, though functional in design, presents certain limitations. It does not account for common compensatory patterns such as elevation of the mouth floor, tongue force, or spatial constraints for the tongue within the maxillary arch, which might have influenced mobility measurements and their functional implications. Notably, the original working-group of Zaghi has since acknowledged these limitations and revised the measurement protocol to address them [17].

#### 4.2.2 Implications of the Kotlow classification for TMD association

Kotlow’s anatomical classification divides free tongue length into five categories. As no patients in the current study presented with complete ankyloglossia, only four subtypes were included in the statistical comparison. Despite the expectation that Kotlow’s more detailed subdivision might weaken the statistical outcome, this classification was the only one that showed a significant correlation with TMD.

It should be noted that Kotlow’s classification was originally validated for children and adolescents up to 14 years of age [14], and the reference values are not adapted for adults. Since the tongue continues to grow beyond adolescence, it is reasonable to assume that the range of normative values increases with age. As a result, the degree of tongue restriction in adult patients may have been underestimated, and the actual correlation with TMD could be even stronger than observed.

Furthermore, although the Kotlow’s classification was validated in pediatric populations, its application in adults may yield stronger correlations due to the anatomical stability achieved after growth. In addition, ankyloglossia may influence muscle activity in reverse, as the functional impairment of speech and swallowing could lead to altered neuromuscular patterns in affected patients.

## 5. Conclusions

This study observed a weak correlation between TMD and the degree of ankyloglossia. To better understand the temporal and potential causal relationship between these conditions, further prospective studies with larger patient cohorts are needed. Future research should incorporate occlusal factors and apply a commonly accepted, validated assessment tool specifically adapted for adults, encompassing both anatomical and functional criteria.
